# Supplementary material for: Empirical methods for controlling false positives and estimating confidence in ChIP-Seq peaks
Source: BMC Bioinformatics. 2008 Dec 5;9:523. doi: 10.1186/1471-2105-9-523 (PMC2628906; doi:10.1186/1471-2105-9-523)
Supplement: Additional File 1 — A variety of html documents from the USeq web site detailing the available applications, their best usage, output file type descriptions, command line menus, etc. [file 1471-2105-9-523-S1.zip › USeqUserGuides/JavaDoc.zip!/JavaDoc/bioroot/package-tree.html]

bioroot Class Hierarchy


|  |  |  |
| --- | --- | --- |
| **Overview** \| **Package** \| Class \| Use \| **Tree** \| **Deprecated** \| **Index** \| **Help** | |  |
| PREV   **NEXT** | **FRAMES**    **NO FRAMES**     **All Classes** |  |


---


## Hierarchy For Package bioroot

**Package Hierarchies:**: All Packages

---

## Class Hierarchy

- java.lang.Object
  - bioroot.**BlastMatcher** (implements java.lang.Comparable<T>)- bioroot.**CellTypeBean**- bioroot.**ContactBean**- bioroot.**CronArchive**- bioroot.**DBUtil**- bioroot.**DeleteMeNow**- bioroot.**Feature** (implements java.lang.Comparable<T>)- bioroot.**GeneBean**- javax.servlet.GenericServlet (implements java.io.Serializable, javax.servlet.Servlet, javax.servlet.ServletConfig)
                    - javax.servlet.http.HttpServlet (implements java.io.Serializable)
                      - bioroot.**AccountManagement**- bioroot.**Archive**- bioroot.**CellType**- bioroot.**Contact**- bioroot.**Gene**- bioroot.**Login**- bioroot.**Logout**- bioroot.**Marker**- bioroot.**Organism**- bioroot.**Search**- bioroot.**UserRegistration**
                    - bioroot.**IdNameMatch**- bioroot.**LabGroup**- bioroot.**LoginBean**- bioroot.**MarkerBean**- bioroot.**OrganismBean**- bioroot.**Organization**- bioroot.**PatternMatch**- bioroot.**Preference**- bioroot.**ReagentBean** (implements java.lang.Comparable<T>)- bioroot.**SearchBean**- bioroot.**SearchHit** (implements java.lang.Comparable<T>)- bioroot.**Spammer**- bioroot.**TableHeading** (implements java.lang.Comparable<T>)- bioroot.**UserBean**- bioroot.**UserRegistrationBean**- bioroot.**Util**

---


|  |  |  |
| --- | --- | --- |
| **Overview** \| **Package** \| Class \| Use \| **Tree** \| **Deprecated** \| **Index** \| **Help** | |  |
| PREV   **NEXT** | **FRAMES**    **NO FRAMES**     **All Classes** |  |


---
